# Rodent activity in municipal waste collection premises in Singapore: an analysis of risk factors using mixed-effects modelling

**DOI:** 10.1038/s41598-023-29405-2

**Published:** 2023-02-21

**Authors:** Stacy Soh, Chee Heong Chua, Zhi Wei Neo, Marcella Kong, Bee Leng Ong, Joel Aik

**Affiliations:** 1 Environmental Health Institute, National Environment Agency, 40 Scotts Road, Environment Building, #13-00, Singapore, 228231 Singapore; 2 Environmental Public Health Operations Group, National Environment Agency, 40 Scotts Road, Environment Building, #13-00, Singapore, 228231 Singapore; 3 Pre-Hospital & Emergency Research Centre, Duke-NUS Medical School, 8 College Road, Singapore, 169857 Singapore

**Keywords:** Population dynamics, Urban ecology

## Abstract

Refuse storage and collection systems are potential sources of food and harbourage areas for rodents which transmit pathogens. We examined the factors associated with rodent activity in public housing municipal waste collection premises in a highly urbanized city-state. We analysed data from April 2019 to March 2020 in mixed-effects logistic regression models to examine the independent factors associated with rodent activity in central refuse chute rooms (CRCs), individual refuse chute (IRC) bin chambers and bin centres. We accounted for within-year patterns, repeated measures and nested effects. We observed a heterogeneous spatial distribution of rodent activity. Rodent droppings were strongly associated with rodent activity in CRCs (aOR: 6.20, 95% CI: 4.20–9.15), bin centres (aOR: 3.61, 95% CI: 1.70–7.64) and IRC bin chambers (aOR: 90.84, 95% CI: 70.13–117.67). Gnaw marks were positively associated with rodent activity in CRCs (aOR: 5.61, 95% CI: 3.55–8.97) and IRC bin chambers (aOR: 2.05, 95% CI: 1.43–2.95), as were rub marks in CRCs (aOR: 5.04, 95% CI: 3.44–7.37) and IRC bin chambers (aOR: 3.07, 95% CI: 1.74–5.42). Each burrow increased the odds of rodent sightings in bin centres (aOR: 1.03, 95% CI: 1.00–1.06). The odds of rodent sightings in an IRC bin chamber increased with every additional bin chute chamber within the same block (aOR: 1.04, 95% CI: 1.01–1.07). We identified several factors that well predicted rodent activity in waste collection premises. Municipal estate managers with limited resources can adopt a risk-based approach in tailoring the focus of their rodent control interventions.

## Introduction

Rodents are reservoirs for zoonoses and play a significant role in the transmission of infectious diseases such as plague, leptospirosis and salmonellosis^1^. Rodents also act as hosts for pathogen carrying arthropod vectors which can transmit diseases such as Rocky Mountain Spotted fever and Lyme disease^2,3^. The commensal rodent species *Rattus rattus (R. rattus)* and *Rattus norvegicus (R. norvigus)*, have been reported with the carriage of pathogens such as *Leptospira interrogans, Rickettsia typhi, Yersinia pestis, Streptobacillus monilliformis*, and Seoul hantavirus^4^. The bacillus *Yersinia pestis* found in these rodents, causes plague, a flea borne zoonotic disease responsible for three widespread pandemics, most notably the Black Death, resulting in millions of lives lost^5^. *R. rattus* and *R. norvegicus* are also the principal source of Leptospira infections, a significant source of morbidity and mortality worldwide responsible for an estimated 1.03 million infections and 58,900 deaths annually^6^. In densely populated South-East Asia, rodent-borne diseases including Leptospirosis, hemorrhagic fever with renal syndrome and rickettsial infections (murine typhus and scrub typhus) have been detected in human populations^7–9^.

*Rattus rattus* and *Rattus norvegicus* are most prevalent in urban environments^10^ where favourable environmental conditions allow for rodent populations to thrive. Urban environments provide a reliable and accessible supply of food^11^ from discarded food waste originating from food establishments and waste collection points such as garbage bins. Urban rats are able to easily seek harbourage in sewer systems, storm drains, infrastructure and create burrows^12,13^. The ease of accessibility to food and shelter provide conducive conditions for persistent rodent infestations^14^. Refuse collection and disposal premises, which provide both an abundance of food and shelter for rodents, have been known to be associated with rodent infestations^15,16^. Poor waste disposal methods, aging infrastructure and dense human populations are among other risk factors that have been consistently associated with rodent infestations^17^.

A number of previous studies have identified infrastructural, environmental and socioeconomic risk factors for rodent infestation. Urban areas with relative lower income levels, higher levels of access, food and harbourage sources were independently associated with higher levels of rodent infestation in Sao Paulo, Brazil^18^. In Kentucky, United States (US), unsecured food and water access in combination with areas of harbourage were positively associated with rodent activity^19^. In Johannesburg, South Africa, lower income levels, dampness and cracks in residences were positively associated with reports of rodent infestation while the presence of a domestic cat was protective against such reports^20^. A study in Salvador, Brazil found that dilapidated fences/walls, and increased proximity to sewer access was associated with higher risks of household rodent infestation^21^. High municipal waste volume and large numbers of residential units have been reported to increase the likelihood of rat infestation in New York, US^22^. While high density housing and older buildings were linked to higher rat activity in the sewer system in Barcelona, Spain^23^. To the best of our knowledge, no studies have assessed the factors associated with rodent activity in densely populated high-rise residential housing estates in South-East Asia.

We hypothesized that rodent activity in waste collection premises in public housing estates could be determined by visual cues such as rodent droppings, gnaw marks and rub marks. We also hypothesized that rodent activity in these estates would increase with more sources of food contributed by retail food establishments. In this study, we aimed to assess the factors associated with rodent activity in waste collection premises—an important potential source of food and harbourage for rodents. Our objective was to identify factors that could positively predict rodent activity in these premises, in order to inform the adoption of estate-specific risk-based approaches in rodent control measures for urban, high density public housing estate managers in Singapore.

## Methods

### Ethics statement

This study was granted approval by the Environmental Health Institute of the National Environment Agency, Singapore (TS271). The study did not involve human participants. No rodents were trapped nor harmed in this study.

### Study area

Singapore is a cosmopolitan city-state situated in the tropics. Home to an estimated population of 5.7 million within a land area of 719 sq km, it is among the densest cities in the world. More than 80% of its residents live in public residential estates built by the government^24^. Most public residential apartment blocks are between 10 and 30 storeys high, with some reaching 40–50 storeys. In each public residential estate, a town council is appointed to provide municipal services that include estate management, cleaning and waste collection. At the time of this study, there were 16 town councils.

### Central refuse chutes, bin chutes and bin centres

All high-rise public housing apartment blocks in Singapore were designed with waste collection systems that channel municipal waste from each floor to the ground floor where it is aggregated for removal by waste collectors.

#### Central refuse chute (CRC) rooms

**Figure 1:**
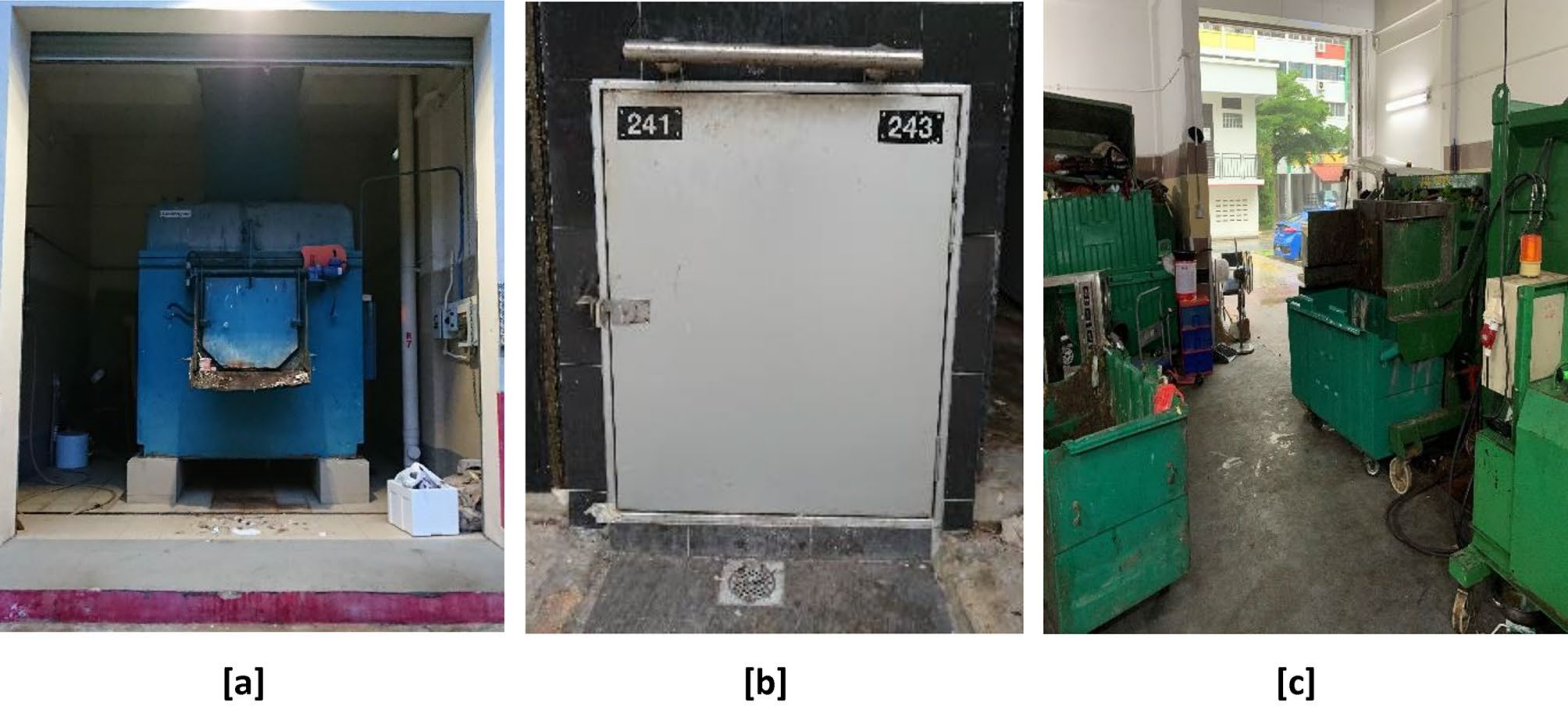
Waste collection premises in public housing estates in Singapore. (**a**) Central refuse chute room, (**b**) individual refuse chute bin chamber and (**c**) bulk waste bins within a refuse bin centre.

Public apartment blocks built after 1989 were designed with the central refuse chute (CRC) system. Household waste is disposed through a refuse chute hopper located in the shared common space of every residential floor. The CRC system channels waste into a compactor located in a purpose-built CRC room on the ground floor of the building (see Fig. 1a). Household waste is collected from the purpose-built room by waste collection trucks daily.

#### Individual refuse chute (IRC) bin chambers

Public apartment blocks built between the 1960s and 1989 were designed with the individual refuse chute (IRC) system. Household waste is disposed through a refuse chute hopper located within each home. A bin chute channels the waste from each floor into an open top bin located in the refuse bin chamber on the ground level. Depending on the design, each public residential apartment block may have between 8 and 12 bin chutes. Sole access to the refuse bin chamber is through a metal door (see Fig. 1b). Refuse from the open top bins are manually transferred into electrically powered refuse carts daily by workers employed by each town council.

#### Bin centres

The electrically powered carts transport refuse to a bin centre located within the same neighbourhood. There, municipal waste from different apartment blocks is aggregated into bulk bins and are subsequently removed daily by waste collection trucks. The main access to a bin centre is through a manual gate or electrically power roller shutter which extends to the floor (see Fig. 1c).

### Rodent surveillance

The National Environment Agency (Singapore) is the national regulatory authority on the control of vector-borne diseases. As part of its surveillance programme, the agency conducts regular inspections of CRC rooms, IRC bin chambers and bin centres in public housing estates with the aim of identifying areas where rodent control measures need to be intensified. Inspections are conducted by a team of surveyors with certification and professional experience in vector control. No rodents are trapped because the primary motivation for the inspections was surveillance. Inspection findings are then communicated to each municipal town council to inform their control activity plans. CRC rooms, IRC bin chambers and bin centres are typically scheduled for bi-monthly inspection cycles by the NEA, though some premises may not be inspected on time due to other priorities for vector control. In addition to these premises, the NEA also identifies rodent burrows in these housing estates. Information on the conditions of CRC rooms, IRC bin chambers and bin centres as well as rodent burrows are communicated to the appointed town councils for the purpose of prioritizing rodent control measures. We obtained all national-level inspection records of CRC rooms, IRC bin chambers, bin centres and rodent burrows from the NEA from April 2019 to March 2020.

### Outcome measure

In this study, the outcome of interest was live rodent sightings. We assessed the odds of rodent activity by creating a dependent variable which was coded with a value of “1” to indicate the sighting of at least one rodent and with “0” if there were no sightings. This modelling approach allowed us to overcome measurement error resulting from the inaccurate enumeration of rodents, since higher counts of rodent sightings were less reliable compared to lower counts due to the greater potential for double-counting.

### Municipal waste collection premises

All inspection records contained information on the date and postal address of each CRC room, IRC bin chamber and bin centre that was inspected. The conditions of each of the waste collection premises were also documented in each inspection record. This included the presence of active signs of rodent activity: (1) rodent droppings, (2) gnaw marks and (3) rub marks within CRCs and bin centres. We coded these independent binary variables with a value of “1” to indicate any positive reports of these observations and “0” if they were absent.

### Rodent burrows and licenced food establishments

In Singapore, rodent burrows are one of several indicators for assessing the level of rodent infestation. Areas with higher counts of rodent burrows are prioritised for further investigation and more concerted pest control efforts. In a systematic review of the behaviour and home range of *Rattus norvegicus* and *Rattus rattus* in urban cities, it was reported that the daily movement of these rodents were usually between 30 and 150 m^25^. Licenced food establishments may support rodent population activity if their food storage and food waste management practices are inadequate. Referencing the postal address of each CRC room, bin centre and each apartment block with IRCs, we obtained the number of rodent burrows and restaurants within a 150 m radius. We incorporated the respective number of these premises as continuous variables in our analysis.

### Statistical analysis

We analysed the dependence of rodent sightings in CRC rooms, bin centres and IRC bin chambers on various independent factors in three separate logistic regression models. The location of each CRC and bin centre was uniquely defined by its postal address, while the location of each bin chute was uniquely defined by the apartment block postal code as well as the chute unit number. Using postal codes, we allowed for random effects to account for correlations among repeated measures at each location. As each of these locations were nested within each public residential estate, we also allowed for random effects at the town council level and at the block level for IRC bin chutes. We simultaneously included all independent variables within the final models for CRC rooms (Eq. 1), bin centres (Eq. 2), and IRC bin chambers (Eq. 3) which are described here:

1 $$\mathrm{log}\left({Y}_{ij}\right)={\beta }_{0} + {\beta }_{1}droppings + {\beta }_{2}gnaw + {\beta }_{3}rub + {\beta }_{4}burrows + {\beta }_{5}restaurant +{\beta }_{z}month +{\alpha }_{i} + {\varepsilon }_{ij}$$

2 $$\mathrm{log}\left({Y}_{ij}\right)={\beta }_{0} + {\beta }_{1}droppings + {\beta }_{2}gnaw + {\beta }_{3}rub + {\beta }_{4}burrows + {\beta }_{5}restaurant +{\beta }_{z}month +{\alpha }_{i} + {\varepsilon }_{ij}$$

3 $$\mathrm{log}\left({Y}_{ij}\right)={\beta }_{0} + {\beta }_{1}droppings + {\beta }_{2}gnaw + {\beta }_{3}rub + {\beta }_{4}burrows + {\beta }_{5}restaurant+ { {\beta }_{6}IRC bin chambers +\beta }_{z}month +{\alpha }_{i} + {\varepsilon }_{ij}$$

where *Y* denotes the observation of at least one rodent within the inspected premises located at *i* on at each inspection cycle *j*. β_0_ is the intercept for *Y*. The binary variables *droppings, gnaw* and *rub* refer to observations of the presence of rodent droppings, gnaw marks and rub marks respectively. The continuous variable *restaurant* refers to the number of licenced eating establishments within a 150 m radius from the reference postal address. *IRC bin chambers* refer to the remaining counts of IRC bin chambers within the same block. β_1_ to β_6_ refer to the estimated coefficients of the respective variables. We included the variable *month* which refers to the calendar month in which the inspection was carried out in order to account for potential within-year variations in the outcome measure. β_z_ refers to the estimated coefficients for the effects of the respective month *z*. α_i_ represents the random effects for each premises with repeated measurements while ε_ij_ represents the random effects of the measurements nested within each town council. In exploratory analysis, we examined whether food establishments modified the relationship between rodent burrows and rodent activity. We assessed statistical significance at the 5% level. All analyses were conducted in Stata 12 (StataCorp LP) and ArcGIS 10.5.1.

### Ethics approval

This study was granted approval by the Environmental Health Institute of the National Environment Agency, Singapore (TS271). The study did not involve human participants. No rodents were trapped nor harmed in this study.

## Results

### Descriptive statistics

The highest number of rodent sightings as a proportion of inspections occurred in refuse bin centres (1.4%), followed by IRC bin chambers (0.9%) and then CRCs (0.7%). When we considered ever-rodent sightings without regard to the number of inspections, 2.9% (n = 170) of residential blocks with CRCs had at least one positive report of rodent sightings while 4.6% (n = 265) of residential blocks with IRC bin chambers had at least one positive report of rodent sightings. Rodent droppings were observed more frequently compared to rub marks and gnaw marks in CRCs and bin centres (Table 1). The mean number of rodent burrows in the vicinity of bin centres was more than tenfold that around CRCs.

**Table 1:** Characteristics of central refuse chutes, individual refuse chute bin chambers and bin centres in Singapore, 2019 to 2020.

Waste collection premises	CRCs (n = 5817)	IRC bin chambers (n = 44,337)	Bin centres (n = 796)
Number (% of inspections)			
Rodent sightings	210 (0.7)	524 (0.3)	43 (1.4)
Rodent droppings	4626 (16.1)	2274 (1.3)	887 (29.2)
Gnaw marks	1211 (4.2)	6590 (3.8)	371 (12.2)
Rub marks	2929 (10.2)	407 (0.24)	374 (2.3)
Mean (95% CIs)			
Rodent burrows	0.3 (0.30–0.31)	0.57 (0.57–0.58)	4.15 (4.07–4.22)
Restaurants	0.56 (0.55–0.57)	1.14 (1.13–1.14)	0.40 (0.38–0.43)
No. of inspections	4.95 (4.90–4.99)	3.91 (3.89–3.92)	3.81 (3.69–3.93)

**Figure 2:**
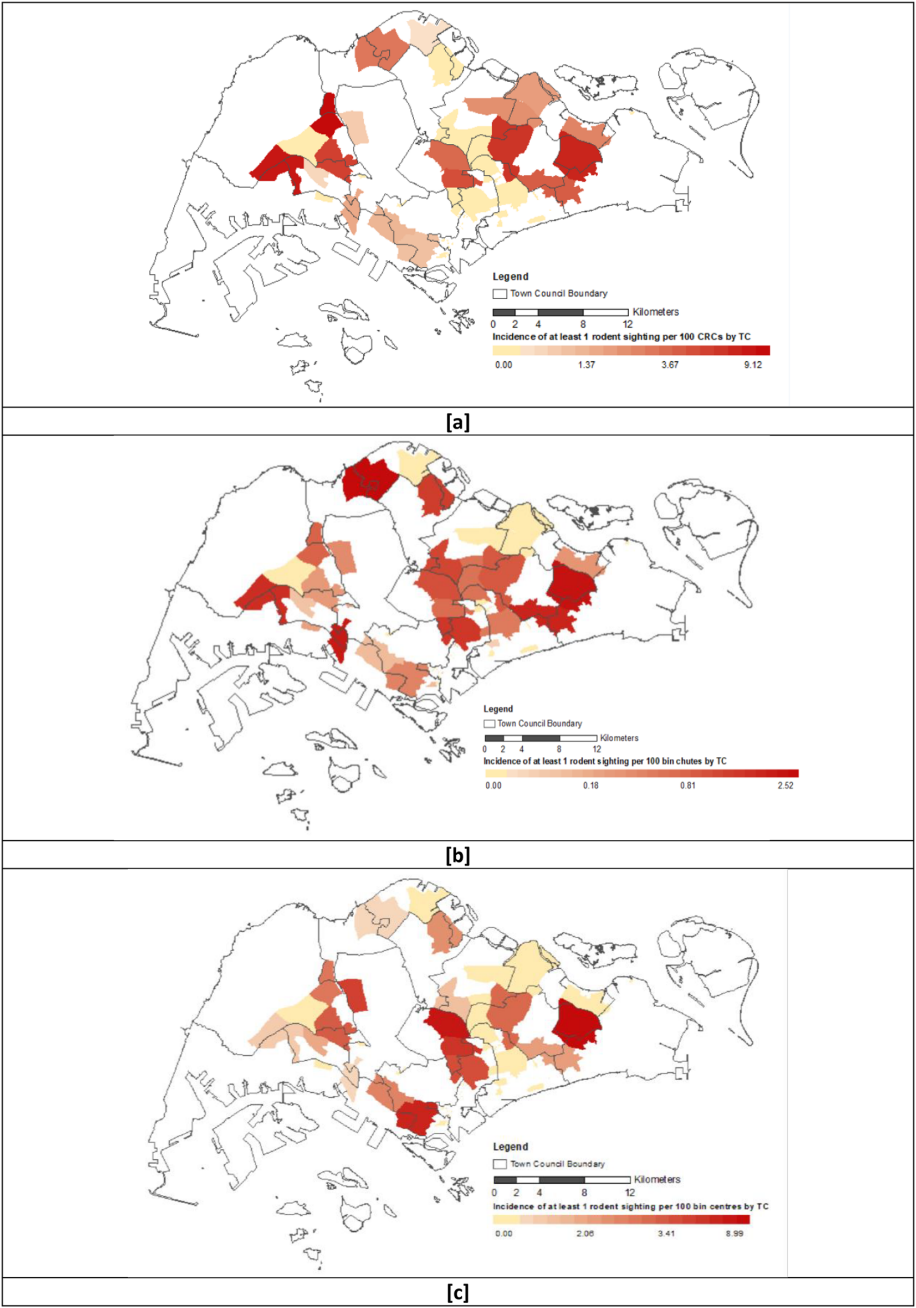
Spatial Incidence of Rodent Sightings in Singapore, 2019–2020. The coloured polygons represent the incidence of rodent sightings in CRCs (**a**), IRC bin chambers (**b**) and bin centres (**c**) per 100 premises in each town council-managed housing estate. The colour density is positively related to the incidence of rodent sightings^26^.

Rodent sightings were heterogeneously distributed across public housing estates. Higher rodent activity in CRCs was observed in some estates located in the eastern and western sectors of Singapore (see Fig. 2a) and in IRC bin chambers in estates across the island (see Fig. 2b). Rodent activity in bin centres in some housing estates in the central, southern and eastern areas were higher than others (see Fig. 2c).

### Regression analysis

#### CRCs

**Figure 3:**
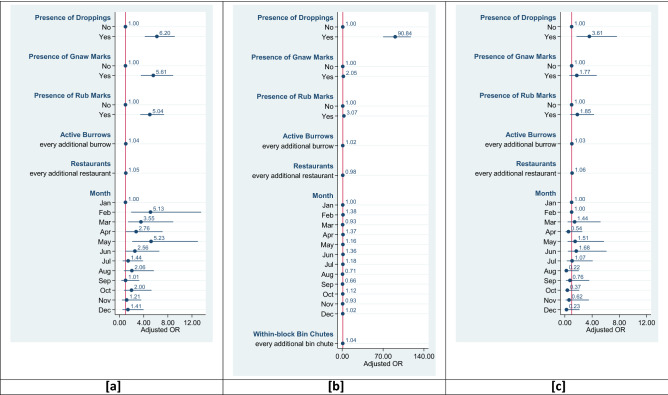
Adjusted ORs for factors associated with reported rodent sightings in (**a**) CRC rooms, (**b**) Individual Refuse Chute Bin Chambers and (**c**) Bin Centres. The solid circles represent the point estimates, and the horizontal navy blue lines indicate the 95% confidence intervals for those estimates. The vertical red line indicates the null value of 1.00.

The odds of rodent sightings were higher in CRC rooms which had other visual signs of rodent activity such as the presence of rodent droppings (aOR: 6.20, 95% CI: 4.20–9.15), rodent gnaw marks (aOR: 5.61, 95% CI: 3.55–8.97) and rub marks (aOR: 5.04, 95% CI: 3.44–7.37) compared to those that did not have these signs (see Fig. 3a). The number of licenced restaurants and rodent burrows both were positively associated with rodent sightings but we could not rule these out as chance findings (p > 0.05). The relationship between burrows and rodent sightings was not altered by the presence of restaurants (p = 0.986). We observed some evidence of within-year variations, with increased odds of rodent sightings in the months spanning February to May relative to January (see Fig. 3a).

#### Individual refuse chute bin chambers

Rodent droppings were positively associated with rodent sightings in IRC bin chambers (aOR: 90.84, 95% CI: 70.13–117.67) as were rodent gnaw marks (aOR: 2.05, 95% CI: 1.43–2.95) and rub marks (aOR: 3.07, 95% CI: 1.74–5.42) (see Fig. 3b). The number of rodent burrows both was positively associated with rodent sightings but we could not rule this out as chance findings (p > 0.05). The relationship between burrows and rodent sightings was not altered by the restaurants (p = 0.250). The odds of rodent sightings increased with every additional bin chute chamber within the same block (aOR: 1.04, 95% CI: 1.01–1.07). The odds of rodent activity appeared to be slightly higher in the first half of the year, though they were non-significant (see Fig. 3b).

#### Bin centres

The odds of rodent sightings were higher in bin centres which had rodent droppings (aOR: 3.61, 95% CI: 1.70–7.64) compared to those that did not. Each active burrow increased the odds of rodent sightings (aOR: 1.03, 95% CI: 1.00, 1.06) (see Fig. 3c). Rodent gnaw marks, rub marks, and the number of licenced restaurants did not have any significant influence on rodent sightings. The relationship between burrows and rodent sightings was not altered by the restaurants (p = 0.189). The odds of rodent activity were higher in the months of March, May, June and July, though they were non-significant (see Fig. 3c).

## Discussion

Commensal rodents serve as important reservoirs of rodent-borne pathogens. Efforts to reduce the risk of pathogen transmission include decimating rodent populations, altering access pathways, upholding good waste management practices and denying easy access to food sources. In our study, we examined the incidence of rodent activity in waste collection premises in public residential estates in Singapore and examined the factors associated with rodent activity to inform the priority of rodent control measures of resource limited municipal estate managers.

Of the three types of waste collection premises, rodent activity had the highest incidence in refuse bin centres followed by CRCs and IRC bin chambers. Refuse bin centres are prone to refuse spillage because refuse is manually transferred from refuse collection carts into bulk bins and refuse compactors located within the centres. Bin centres tend to be larger than CRCs and IRC bin chambers and the storage of bulky waste that provide additional areas of rodent harbourage are a common sight in Singapore. IRC chambers and refuse bin centres in combination far outnumber CRCs, and the former two are a distinct characteristic of older public housing estates in Singapore. This suggests that older public housing estates have a higher propensity for rodent infestation compared to newer ones. Aging infrastructure can also provide a greater number of harbourage areas and alternate access pathways for rodent travel that increase their ability to obtain food sources. Our study findings were in support of previous studies which found that older infrastructure was associated with a greater likelihood of rodent activity^22,23^.

We also found that the number of IRC bin chutes was positively associated with rodent infestation. Fluids from food waste in IRC bin chambers are drained directly into a sanitary line that is common to all other bin chambers within the same building. A possible explanation therefore is that rodents which find their way into the sanitary line can easily access all bin chambers in the same building. This suggests that preventing individual bin chamber access may reduce food availability to rodents which traverse the sanitary line in search of food sources.

In the present study, we observed that rodent sightings were relatively higher in some months in the first half of the calendar year compared to the second half. Even though our estimates were positive, those for some months were not statistically significant. In Singapore, end-December, January to February are usually associated with increased food production due to the year-end (Christmas and New Year celebrations) and early-year (Chinese New Year) festivities. A proportionate increase in food waste over that period could improve survivability of rodents that leads to increased mating and reproduction. We therefore postulate that the higher seasonal rodent activity is plausible but recommend that future studies be conducted with sufficient longitude to examine the differences in the seasonal pattern across the three categories of premises more closely. A previous study in Harbin, China^27^ reported a seasonal pattern in the age composition of *R. norvegicus* while an ecological study on *R. norvegicus* in Salvador Brazil did not find any difference in the number of rats trapped between the dry and rainy seasons^28^. The inconsistent seasonal findings between studies could be due to the differences in the climate, degree of urbanization and environmental conditions of study locations.

The relative rise in rodent activity in the first half of the year coupled with older estates being at greater risk of rodent activity suggest that municipal town councils which prioritize regular infrastructural repairs and improvements in older estates and complete them in the second half of each calendar year would help mitigate the anticipated rise in the first half of the new calendar year.

In our study, we examined the relations between visual cues and rodent activity to help estate managers prioritise their control efforts. We found that rodent droppings were a common positive predictor of rodent activity across all three categories of waste collection premises. In particular, the odds of droppings in IRC bin chambers were the highest among the three categories of premises. We hypothesize that the probability of rodent dropping sightings was in part related to the accessibility of food waste and thus time spent by rodents within the respective waste collection premises. Each IRC chamber contains an open top bin that receives waste that is disposed down the IRC chamber chute. Food waste in IRC bin chambers are thus more easily accessed by rodents compared to in CRCs where waste is stored in a compactor and in bin centres where bulk bins are covered until the waste is compacted or collected.

In Salvador, Brazil, the presence of *Rattus norvegicus* droppings were independently associated with an increased risk of *Leptospira* infection in humans^29^. Further research on site-specific *Leptospira* infection risks in Singapore are required to affirm the utility of droppings as an indicator for *Leptospira* infection risk. In addition, rub marks and gnaw marks were also positive predictors of rodent activity in CRCs and IRC bin chambers. A study in Chile reported that gnaw marks and holes, as well as grease or rub marks left behind by rodent travel were indicators of rodent activity^30^. A previous study carried out in an urban city in Taiwan reported that rodent droppings and rub marks were well correlated with rodent infestation^31^. Our findings, which were in support of these previous studies, suggest that estate managers can maximise the cost effectiveness of their resources by focusing their control efforts based on visual cues without relying solely on trapping activities for surveillance.

We found a positive relationship between the number of rodent burrows and rodent activity in all three waste collection premises, though this was only significant for refuse bin centres. That the direction of effect for burrows was consistent these three premises, was a reassuring observation. It is possible that we did not have enough study power to establish the observed positive relations in CRCs and IRC bin chambers. Therefore future studies should seek to confirm our findings. *R. norvegicus* excavate extensive burrow systems that are able to house a large number of rats^32^. They exhibit a strong preference for creating burrows in loose soil and on sloping terrain^33^ and construct shallow burrows in close proximity to water bodies and food sources^34^. As rodent burrows are primarily used for nesting, food storage and harbourage purposes^35^, burrows can provide important information about the extent of rodent activity in an area and may be used as an indicator for estate managers to focus their investigations.

A previous study in New York, United States found that the presence of numerous restaurants, or having older infrastructure were associated with increased levels of *R. norvegicus*^22^*.* Unexpectedly, we did not find any evidence that the number of dining establishments was associated with rodent activity. However, instances of rodent activity have been reported in food establishments in Singapore^36–38^. We hypothesize that rodent movement is restricted to the surrounding area of the food establishments due to the plethora of food available, with little reason for rodents to venture into waste collection premises. Future studies examining the relationship between rodent activity in food establishments and waste collection premises are required to confirm this.

In our study, the presence of gnaw marks (aOR: 5.61), rub marks (aOR: 5.04) in CRCs and rodent droppings in CRCs (aOR: 6.20), IRC bin chambers (aOR: 90.84) and bin centres (aOR: 3.61) had the largest strengths of association with rodent activity. Comparatively, in a study in Johannesburg, South Africa, predictors such as dampness (aOR: 2.54) and cracks (aOR: 1.92) in homes had relatively smaller effects on rodent activity^20^, while a study in Salvador, Brazil found relatively larger effects of homes with dilapidated fences and walls (aOR: 8.95) and those built on earthen slopes (aOR: 4.95)^21^. This suggests that rodent activity can be strongly influenced by site- and setting-specific factors, and supports the body of evidence on the strong adaptability of rodents in our urban environment”.

Urban environments have the capacity to alter the biology of the pathogens, hosts and vectors, which can influence disease transmission^39^. The proximate setting of dense urban environments allows for close contact between humans and synanthropic rodents, thereby increasing the transmission risk of zoonotic diseases^4^. In addition to causing diseases in human populations, urban rats are also known to compromise food safety, damage infrastructure and cause mental health distress^25,40^. The responsibility of rodent control in residential estates is important but may be one among many other competing public health and estate management responsibilities that municipal town councils have to undertake. Consequently, estate managers have to prioritize their limited resources in order to maximise the cost effectiveness of their resource allocation choices. Based on our study findings, we recommend that estate managers adopt a risk-based approach in vector control resource allocation in waste collection premises according to infrastructural age and visual cues for rodent activity.

IRC bin chambers which are a distinct feature of the oldest residential buildings, were observed with a substantially higher odds of rodent activity compared to the other categories of waste collection premises. This suggests that rodent control resource allocation should be prioritized in older residential estates. The clear seasonal pattern of rodent activity in CRCs suggests that estate managers can increase their rodent control activities thereat in the first half of the year. Finally, easy access to food waste directly increases the probability of survival and consequently the rodent population size. Future research should examine the quality of municipal solid waste management and the waste processing flow in residential estates to determine how rodent access to food waste can be further minimized to reduce the population of rodents.

### Study strengths and limitations

We analysed data from all public residential estates in Singapore; our findings are thus generalizable at the national level. The use of outcomes and independent measures from individual waste collection premises over multiple cycles of inspection provided stronger evidence for causal inferences. We analysed data over 12 months to account for within-year variations that could influence the outcome measure. Rodents were visually identified without molecular speciation because no trapping was carried out. Though the majority of rodents were observed to be *Rattus norvegicus*, which is the most common species of rodents in public housing estates in Singapore, we could not rule out misclassification of rodents. However, our findings remain relevant for municipal authorities seeking to prioritize resources for vector control in waste collection premises under their care.

## Conclusion

We found a spatially heterogeneous distribution of rodent activity in public residential estate waste collection premises. Older housing estates may have a higher propensity for rodent activity. Our study findings also identified visual cues and other factors that well predicted higher levels of rodent activity. Municipal town councils with limited resources should adopt a risk-based approach in tailoring the focus of their rodent control interventions in order to reduce rodent activity and consequently rodent-borne disease transmission in residential estates.

## Data Availability

The data is available upon reasonable request from the Environmental Public Health Operations Group, NEA (email: chua_chee_heong@nea.gov.sg).
